# Adiposity in relation to age at menarche and other reproductive factors among 300 000 Chinese women: findings from China Kadoorie Biobank study

**DOI:** 10.1093/ije/dyw165

**Published:** 2016-08-12

**Authors:** Ling Yang, Liming Li, Iona Y Millwood, Sarah Lewington, Yu Guo, Paul Sherliker, Sanne AE Peters, Zheng Bian, Xianping Wu, Min Yu, Huilin Liu, Hongmei Wang, Enke Mao, Junshi Chen, Mark Woodward, Richard Peto, Zhengming Chen, on behalf of the China Kadoorie Biobank study collaborative group (members listed at end of report)

**Affiliations:** 1 Clinical Trial Service Unit and Epidemiological Studies Unit (CTSU), University of Oxford, Oxford, UK,; 2 Chinese Academy of Medical Sciences, Dong Cheng District, Beijing, China,; 3 Department of Public Health, Beijing University, Beijing, China,; 4 The George Institute for Global Health, University of Oxford, Oxford, UK,; 5 Sichuan CDC, Chengdu, Sichuan, China,; 6 Zhejiang CDC, Hangzhou, Zhejiang, China,; 7 Hunan CDC NCDs Prevention and Control Department, Changsha, Hunan, China,; 8 Hainan CDC NCDs Prevention and Control Department, Haikou, Hainan, China,; 9 Maiji CDC, Tianshui, Gansu, China,; 10 China National Center for Food Safety Risk Assessment, Chaoyang District, Beijing, 100021, China and; 11 The George Institute for Global Health, University of Sydney, Australia

**Keywords:** adiposity, reproductive factors, cross-sectional studies, Chinese, women

## Abstract

**Background:** Adiposity is increasing rapidly in China but little is known about the relevance to it of women’s reproductive factors, which differ inter-generationally and from that in the West. We assess associations of adiposity with life-course reproductive factors in Chinese women.

**Methods:** In 2004–08, the nationwide China Kadoorie Biobank recruited 303 000 women aged 30–79 (mean 50) years from 10 diverse regions. Multivariable linear regression was used to examine associations of reproductive factors (e.g. age at menarche/first birth/menopause, parity, breastfeeding and reproductive years) with measures of general [e.g. body mass index (BMI)] and central [e.g. waist circumference (WC)] adiposity in adulthood.

**Results:** Overall, the mean BMI was 23.7 (standard deviation 3.3) kg/m^2^, mean age at menarche was 15 (2) years and nearly all had given birth (99%) and breastfed children (98%). Adiposity was associated inversely with age at menarche and at first birth, with 0.19 and 0.05 kg/m^2^ lower BMI and 0.38 and 0.12 cm lower WC per 1-year delay respectively (*P* < 0.001). Among 128 259 post-menopausal women, adiposity was associated positively with age at menopause and reproductive years, with 0.05 and 0.07 kg/m^2^ higher BMI and 0.12 and 0.17 cm higher WC per 1-year increase, respectively (*P* < 0.001). The proportion with overweight/obesity had similar associations with these reproductive factors. Adiposity had a non-linear positive association with parity, but no association with breastfeeding duration.

**Conclusion:** Among Chinese women, earlier age at menarche and at first birth, later age at menopause and longer reproductive years were independently associated with increased adiposity late in life.

**Key Messages**

- Among 303 000 middle-aged Chinese women recruited in the nationwide China Kadoorie Biobank in 2004–08, the overall mean body mass index (BMI) was 23.7 (standard deviation 3.3) kg/m^2^, mean age at menarche was 15 (2) years and nearly all gave birth (99%) and breastfed children (98%).
- Adiposity was associated inversely with age at menarche and at first birth, with 0.19 and 0.05 kg/m^2^ lower BMI and 0.38 and 0.12 cm lower WC per 1-year delay, respectively.
- Adiposity was associated positively with age at menopause and reproductive years among 130 000 post-menopausal women, with 0.05 and 0.07 kg/m^2^ higher BMI and 0.12 and 0.17 cm higher WC per 1-year increase, respectively.
- Adiposity had a non-linear positive association with parity, but no association with breastfeeding duration.
- The proportion with overweight/obese has similar associations with these reproductive factors.

## Introduction

Greater adiposity is associated with a range of chronic non-communicable diseases, including diabetes, cardiovascular disease (CVD) and certain cancers. Several large studies of mostly Western women have shown that early age at menarche may be associated with increased risk of overweight and obesity.^1^^,^^2^ Several other reproductive factors, such as parity, breastfeeding duration and age at menopause, have also been implicated in adiposity, but evidence is more limited, with inconsistent findings from previous studies.^3–7^ Moreover, few previous studies were able to examine the effects on adiposity of a range of reproductive factors simultaneously and there was a possibility of residual confounding e.g. by smoking or drinking alcohol. Given few Chinese women regularly smoke or drink alcohol compared with Western populations, examining the association between reproductive factors and adiposity in China is particularly interesting.^8^^,^^9^

Reproductive patterns among women in mainland China generally differ from those in Western countries, including much later age at menarche, earlier age at menopause and universal breastfeeding among parous women.^8^ However, these patterns are changing, due partly to the rapid socio-economic developments occurring in China and partly to the introduction of the ‘One Child Policy’ in the late 1970s.^8^ Compared with older generations, women born in more recent decades tend to have a lower mean age at menarche and, particularly in urban areas, lower parity and increased mean age at first birth. Meanwhile, large changes in diet and patterns of physical activity are also taking place in China that may substantially affect the prevalence of overweight and obesity. However, there is still limited evidence about the relationship of adiposity with reproductive history in Chinese women, overall or in different population subgroups.

We report the associations between women’s life-course reproductive factors and adiposity in later life from a large study (the China Kadoorie Biobank study, CKB) that included over 300 000 adult women from 10 diverse areas in China. The main aims of the present study were to examine the associations of general and central adiposity measured in adulthood with age at menarche and several other reproductive factors, both overall and in specific subgroups of participants.

## Methods

### Study population

Detailed information about the CKB study design, methods and participants has been reported previously.^10^^,^^11^ Briefly, the baseline survey took place in 10 geographically defined areas in China during 2004–08 and, in total, 512 891 participants (∼30% of those invited responded), including 302 632 (59%) women aged 30–79 years, were recruited. Trained health workers collected information on general demographic characteristics, socio-economic status, lifestyle habits, women’s reproductive history and medical history using a laptop-based questionnaire; took physical measurements; and collected blood samples for long-term storage. All participants provided written informed consent and local, national and international ethics approval was obtained.

### Assessment of reproductive status

The information on reproductive history included age at menarche, menopause status, parity, oral contraceptive (OC) use and history of hysterectomy or ovarian surgery. For each live birth, data on age at birth and breastfeeding duration were collected. For post-menopausal women, age at menopause was also recorded, which was used subsequently for estimating total reproductive years (i.e. duration between age at menarche and age at menopause).

### Adiposity measures

Anthropometric measurements were taken while participants were wearing light clothes and no shoes. Standing height was measured to the nearest 0.1 cm, using a stadiometer. Weight and body fat percentage (%BF) were measured using a bioelectrical impedance device (TANITA-TBF-300GS; Tanita Corp).^12^ Body mass index (BMI) was calculated as weight (kg) divided by the square of standing height (m). Waist circumference (WC) and hip circumference (HC) were measured using a soft non-stretchable tape. WC was measured midway between the lowest rib and the iliac crest. HC was measured at the maximum circumference around the buttocks. The waist–hip ratio (WHR) was the ratio of WC to HC.^10^

### Statistical analysis

For the present study, 32 920 women who had prior major chronic diseases (i.e. CVD, cancer, COPD), hysterectomy or oophorectomy were excluded. A further 4733 women with inconsistent or implausible data for reproductive history or adiposity were excluded. After these exclusions, 264 979 women remained in the main analyses.

Multivariable linear regression models were used to examine the association of each adiposity measure with reproductive factors, adjusting simultaneously for age, region, education, household income, smoking, alcohol drinking, physical activity [calculated in metabolic equivalent tasks (METs-h/day)^13^] and OC use. The model was further adjusted for age at menarche when investigating the association of adiposity with other reproductive factors. Among parous women, the analyses of age at first birth and breastfeeding duration per child were further adjusted for parity. Analyses of age at menopause and reproductive years were only conducted among post-menopausal women, with additional adjustment for other reproductive factors. In separate analyses, we also examined the association between the number of children and adiposity among 191 431 men to explore whether the association may be due to the biological effects of childbearing.

Least-squares means of each adiposity measure were calculated and plotted against categories of specific reproductive factors. If the association was linear, a straight line was plotted through the categorical estimates, where the slopes were based on the estimates from using continuous values of the reproductive factor. To assess the relative importance of general vs central adiposity in relation to reproductive factors, models of central adiposity measures were adjusted for BMI and, similarly, models of general adiposity were adjusted for WC. All of the analyses used SAS v9.3.

## Results

Among 264 979 women, the overall mean [standard deviation (SD)] age was 50.3 (10.4) years and very few regularly smoked (∼2%) or drank alcohol (∼2%). The mean BMI was 23.7 (3.3) kg/m^2^ and the mean WC was 78.7 (9.2) cm, with 28.7% being overweight (BMI 25.0–29.9 kg/m^2^), 4.3% obese (BMI ≥ 30 kg/m^2^) and 42.9% abdominally obese (WC ≥ 80 cm). Compared with urban women, rural women (57%) were slightly younger, less educated, more physically active, and had lower household income and lower levels of both general and central adiposity (Table 1).

**Table 1.** Baseline characteristics of study participants by area

Characteristics	Overall	Area	
		Rural	Urban
Number of women	264 979	151 332	113 647
Mean (SD) age, years	50.3 (10.4)	49.6 (10.2)	51.3 (10.6)
**Socio-economic and lifestyle factors, % or mean (SD)**			
High school or above	17.7	6.3	33.7
Household income >20 000 CNY/year	40.5	30	55.1
Total physical activity, MET-h/day	20.9 (12.8)	22.8 (13.2)	18.4 (11.9)
Current regular smoker	2.3	2.7	1.7
Weekly regular drinker	2.1	2.1	2.1
Ever use of oral contraceptive pill	9.7	8.6	10.9
**Anthropometric measurements, % or mean (SD)**			
BMI, kg/m^2^	23.7 (3.3)	23.4 (3.3)	24.1 (3.4)
Overweight (BMI 25–29.9 kg/m^2^)	28.7	26.3	31.5
Obesity (BMI ≥ 30 kg/m^2^)	4.3	3.6	5.0
Percentage body fat	31.9 (6.9)	31.5 (7.0)	32.4 (6.7)
Standing height, cm	154.2 (5.9)	153.3 (5.8)	155.5 (5.9)
Height adjusted weight, kg	56.5 (4.5)	55.8 (4.4)	57.3 (4.5)
Waist circumference, cm	78.7 (9.2)	78.1 (9.2)	79.4 (9.2)
Abdominal obesity (WC ≥ 80 cm)	42.9	40.6	45.3
Hip circumference, cm	90.9 (6.6)	89.4 (6.1)	92.9 (6.7)
Waist–hip ratio	0.86 (0.07)	0.87 (0.07)	0.85 (0.07)
**Reproductive factors, % or mean (SD)**			
Age at menarche, years	15.4 (1.9)	15.5 (1.9)	15.3 (2.0)
Early menarche (≤12 years)	5.4	4.6	6.6
Parous women	98.8	99.2	98.1
Numbers of live births	2.2 (1.3)	2.5 (1.3)	1.8 (1.2)
Parity >2 children	31.5	38.4	22.3
Age at first birth, years^a^	23.4 (3.1)	22.3 (2.6)	24.7 (3.2)
Never breastfed^a^	2.7	1.4	4.5
Months of breastfeeding per child^a^	15.0 (7.2)	16.7 (7.7)	12.6 (5.8)
Post-menopause at baseline	48.4	49.2	47.4
Age at menopause, years^b^	48.5 (4.1)	48.2 (4.2)	48.9 (3.9)
Reproductive years^b^	32.6 (4.4)	32.2 (4.5)	33.1 (4.3)

^a^Among parous women only; ^b^among post-menopausal women only. BMI, body mass index; SD, standard deviation; WC, waist circumference.

The overall mean age at menarche was 15.4 (1.9) years and only 5.4% women had an early menarche (i.e. ≤12 years), with the proportion higher among younger than older generations (11.1% among those born after 1950 vs 4.0% born before 1950), except for women born in 1940–45, who experienced the great famine around puberty. Almost all women (98.8%) were parous, with 2.2 (1.3) live births on average. The proportion of nulliparous women was higher in urban than rural areas (1.9% vs 0.8%) and among younger generations, particularly in urban areas (e.g. 6% in those born during the 1970s vs 1% born during the 1950s). Among parous women, the mean age at first birth was 23.4 (3.1) years and breastfeeding was almost universal (97.3%), lasting on average 15.0 (7.2) months per child or 35.2 (29.2) months in total. At baseline, 48.4% women reported having a natural menopause, with mean age at menopause of 48.5 (4.1) years and total reproductive years of 32.6 (4.4). The proportion experiencing late menopause (i.e. >53 years) was higher in younger generations, particularly in urban regions (Supplementary Figure 1, available as Supplementary data at *IJE* online). Women with later age at menarche, more childbearing, earlier age at first birth, longer breastfeeding duration, earlier age at menopause and shorter total reproductive years tended to be born in more recent decades, resident in urban areas, be highly educated or with higher household income, but with no clear patterns on smoking, alcohol drinking and physical activity. The above lifestyle factors also varied by adiposity, with those who had higher BMI and WC more likely to be urban and with lower education levels (Supplementary Table 1, available as Supplementary data at *IJE* online).

**Figure 1.**
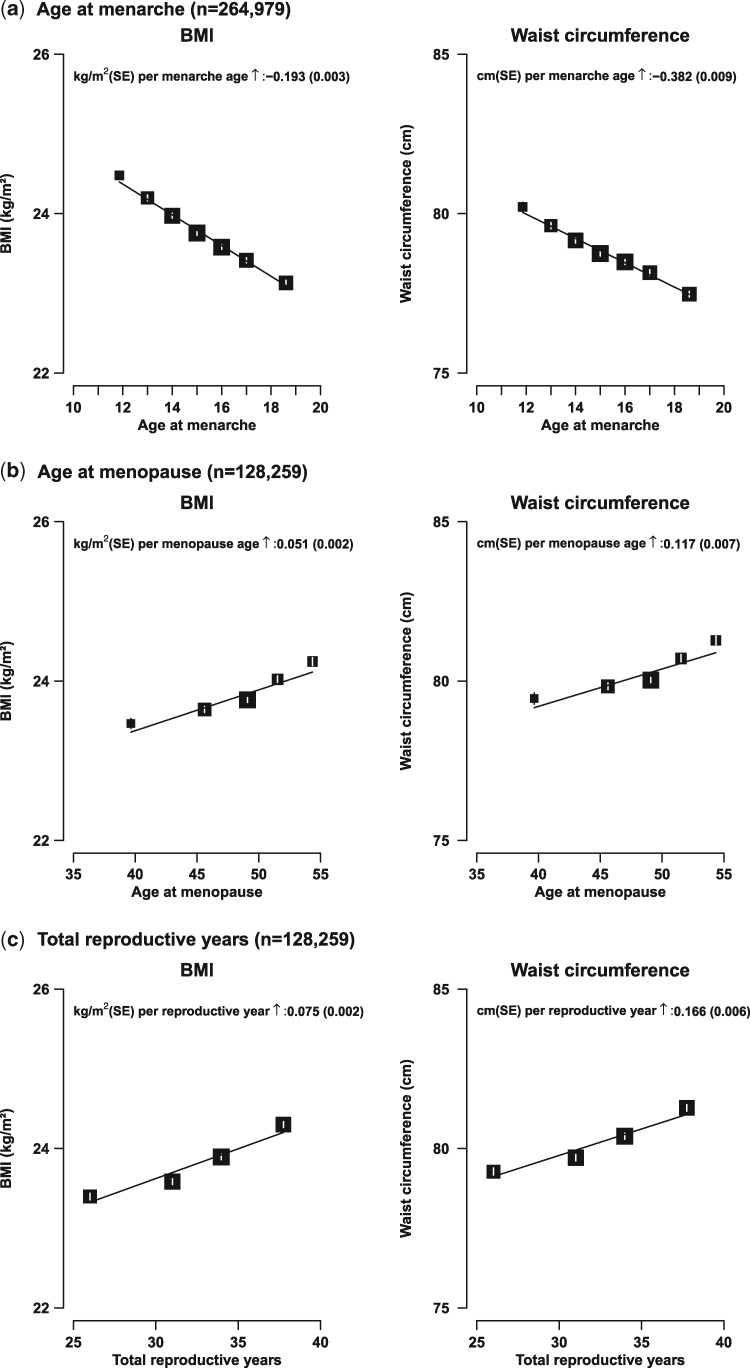
Body mass index (BMI) and waist circumference (WC) vs age at menarche among all women, age at menopause and total reproductive years among post-menopausal women only. The means of each anthropometric measure was adjusted for age, region, education, household income, smoking, alcohol and physical activity. Analyses of age at menopause and reproductive years were only conducted among post-menopausal women, with additional adjustment for other reproductive factors. Closed squares represent the means of anthropometric measures with area inversely proportional to the variance of the mean and the vertical lines represent the corresponding 95% confidence intervals.

Adiposity, irrespective of how it was measured, was associated inversely with age at menarche. The associations appeared to be linear and the corresponding increases in BMI, %BF, WC and WHR per 1 year of earlier onset of menarche were 0.19 kg/m^2^, 0.33%, 0.38 cm and 0.002, respectively (all *P* < 0.001; Figure 1 and Supplementary Table 2, available as Supplementary data at *IJE* online). Early menarche (<12 vs ≥12 years) was associated with 0.7 kg/m^2^ higher BMI (24.4 vs 23.7 kg/m^2^) and 1.5 cm higher WC (80.1 vs 78.6 cm) (*P* < 0.001) (Supplementary Table 2, available as Supplementary data at *IJE* online).

Among 128 259 post-menopausal women, there were positive and apparently linear associations of adiposity with age at menopause and total reproductive years. For each year of delay of age at menopause, BMI and WC increased by 0.05 kg/m^2^ and 0.12 cm, respectively (both *P* < 0.001; Figure 1b). Similarly, compared with women who had menopause before age 53 years, those who experienced menopause later than age 53 had higher mean BMI (24.2 vs 23.8 kg/m^2^) and WC (81.3 vs 80.0 cm) (both *P* < 0.001; Supplementary Table 2, available as Supplementary data at *IJE* online). Likewise, each extra total reproductive year was associated with 0.07 kg/m^2^ higher BMI and 0.17 cm higher WC (both *P* < 0.001; Figure 1c).

**Figure 2.**
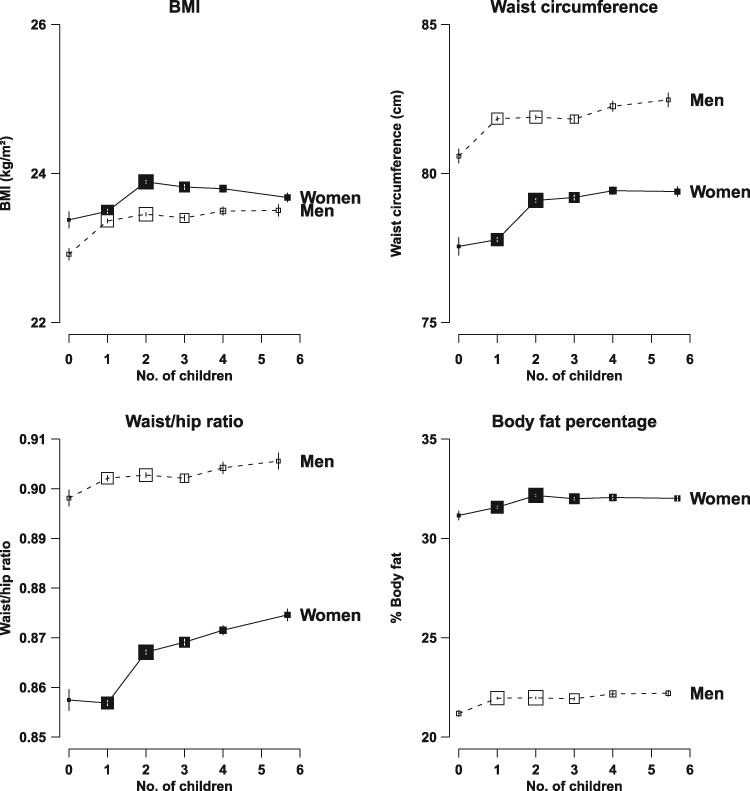
Body mass index (BMI) and waist circumference (WC) vs number of live births in women and number of children fathered in men. The means of each anthropometric measure was adjusted for age, region, education, household income, smoking, alcohol and physical activity. Closed squares and solid lines represent the means of anthropometric measures for women whereas open squares and dash lines represent the means for men. Other conventions as in Figure 1.

Compared with parous women, nulliparous women had modestly lower adiposity but the differences were only significant in urban women and in those with higher levels of education (Supplementary Tables 2–4, available as Supplementary data at *IJE* online). Among parous women, adiposity tended to increase with number of parity, although not in a linear fashion. Among women who had one child, the adjusted mean BMI and WC were 23.5 kg/m^2^ and 77.8 cm, respectively, increasing to 23.9 kg/m^2^ and 79.1 cm in women with two children, beyond which there was little further increase (Figure 2). Similar patterns were also seen for other adiposity measures (Supplementary Table 2, available as Supplementary data at *IJE* online). Likewise, similar associations between adiposity and number of children were also found in men. Compared with men who fathered one or more children, those who had no children had slightly lower BMI (22.9 vs 23.4 kg/m^2^) and WC (80.6 vs 81.9 cm). As in women, among men with children, a similar non-linear positive association was observed between various adiposity measures and increasing number of children (Figure 2).

**Figure 3.**
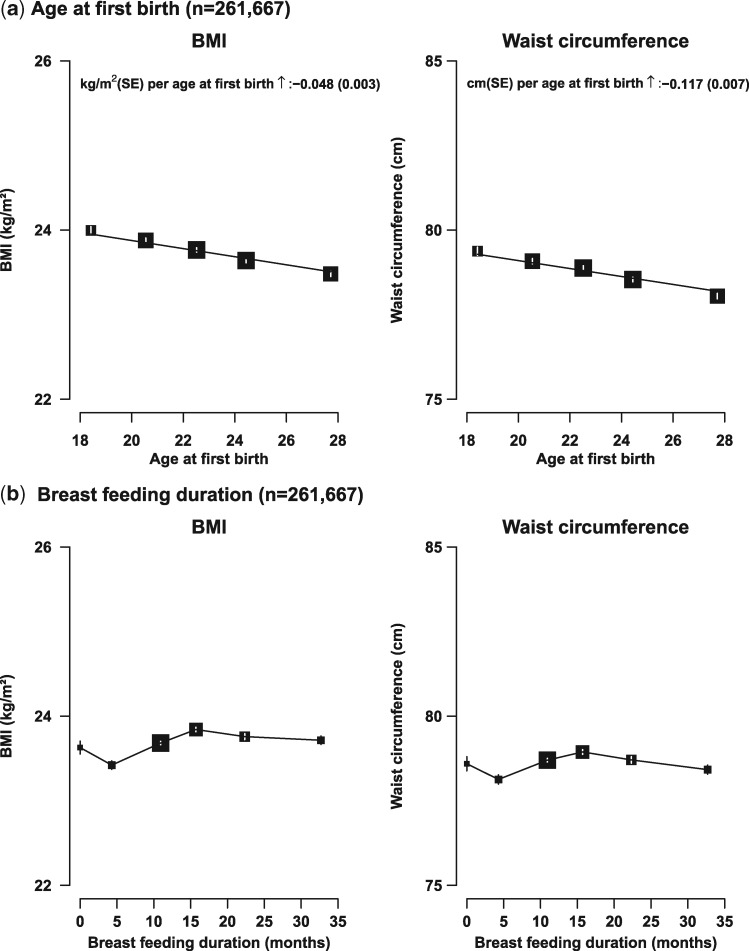
Body mass index (BMI) and waist circumference (WC) vs age at first birth and breastfeeding duration per child among parous women only. Conventions as in Figure 1 and the analyses were further adjusted for age at menarche and parity.

Adiposity was associated inversely and significantly, in a linear fashion, with age at first birth among parous women; each 1 year earlier at first birth was associated with about 0.05 kg/m^2^ higher BMI and 0.12 cm higher WC (Figure 3a; both *P* < 0.001). Parous women who had never breastfed had similar mean BMI and WC to those who had ever breastfed. However, among women who ever breastfed, both BMI and WC increased with breastfeeding duration up to about 18 months per child, beyond which the levels of adiposity plateaued and, if anything, tended to decrease slightly with increasing duration (Figure 3b). The associations between total lactation duration and adiposity were similar to those using breastfeeding months per child as the exposure (data not shown).

After simultaneous adjustment for WC, the associations with reproductive factors observed above remained significant for BMI, although attenuated. In contrast, additional adjustment for BMI eliminated almost completely the associations for WC (Supplementary Table 2, available as Supplementary data at *IJE* online).

Similar patterns of associations were also seen for proportion with abnormal adiposity (overweight/obese and central obesity) vs reproductive factors examined above (Supplementary Figures 2 and 3, available as Supplementary data at *IJE* online). For example, comparing women who had menarche <12 years vs those with menarche >18 years, the proportions of overweight and central obesity were 41% vs 28% and 50% vs 38%, respectively.

**Figure 4.**
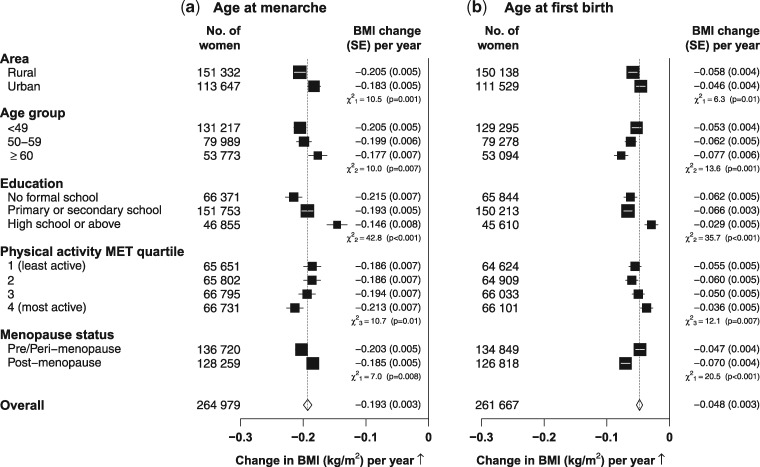
Change in BMI associated with 1-year increase of (a) age at menarche among all women and (b) age at first birth among parous women only in certain population subgroups. The analysis was adjusted for age, area and other lifestyle factors (where appropriate). Each closed square represents a change in BMI per year increasing. The dotted vertical line indicates the overall change in BMI; the open diamond indicates it and its 95% confidence interval.

The pattern of associations (e.g. the direction and the linearity) between adiposity and several other reproductive factors appeared to be generally similar in each subgroup of participants defined by residential area (Supplementary Table 3, available as Supplementary data at *IJE* online), menopausal status (Supplementary Table 4, available as Supplementary data at *IJE* online) or educational level (Supplementary Table 5, available as Supplementary data at *IJE* online). However, the strength of association between adiposity and age at menarche and age at first birth varied somewhat across subgroups of women (Figure 4). For example, the association of adiposity with age at menarche appeared to be somewhat greater among rural women, at younger ages, among those with poor education or who were more physically active or pre-menopausal (P*_heterogeneity_* < 0.01). Among post-menopausal women, however, the associations of adiposity with age at menopause or reproductive years did not appear to vary much in subgroups of participants (data not shown).

## Discussion

In this large study of relatively lean adult women from the general communities of 10 diverse regions in China, adiposity, irrespective of how it was measured, was associated inversely with age at menarche and age at first birth and positively with parity, age at menopause and total reproductive years.

Our findings of increased adiposity with early menarche were consistent with findings in other studies in both Western and Chinese women.^1^^,^^6^^,^^7^^,^^14–17^ In a meta-analysis of 10 studies including 246 671 women, mostly from Western populations,^2^ early menarche (<12 vs ≥12 years) was associated with 0.34 kg/m^2^ higher BMI—an effect only about half as strong as in the present study and in previous small studies in China,^6^ in which the mean BMI was much lower than in the Western populations. It is possible that the effect of earlier age at menarche on adult adiposity may be relatively smaller among girls who were already obese in childhood. Childhood adiposity is much higher in Western populations than in China, which may influence age at menarche and could attenuate the strength of the association observed with adult adiposity.^2^

In this Chinese population, we found that adiposity was positively associated with parity, but not with breastfeeding duration. Similar patterns of non-linear positive association between number of children and adiposity observed in women and men suggested that factors related to childbearing (e.g. stress or lifestyle changes), rather than biological effects, may account for the observed associations. Similar findings were also reported among the 273 478 women in the UK Biobank.^7^ The null association between breastfeeding and adiposity found in the present study, however, differed from those previously observed in Western studies.^4^^,^^5^^,^^16^^,^^18^ In the UK Million Women Study (MWS) including 740 000 post-menopausal women, BMI was associated positively with parity but inversely with breastfeeding duration. At each parity level, the mean BMI was significantly lower among women who had breastfed than those not, with 0.22 kg/m^2^ lower BMI for every 6 months of breastfeeding.^4^ Compared with CKB women, MWS women were on average 7 years older and had higher mean BMI (26.2 vs 23.7 kg/m^2^). Moreover, the proportions of nulliparous women (13% in MWS vs 1% in CKB) and those breastfeeding (69% vs 97%) as well as duration of breastfeeding per child (3 months vs 15 months) also differed significantly between these two study populations. Similarly large differences in patterns of reproductive factors also exist across other studies, which may contribute, at least partly, to the discrepant relationships between parity, breastfeeding and adiposity observed between studies^16^^,^^18^^,^^19^ or between different populations within the same cohort, such as stronger associations found in White than in Black women within a same US cohort.^5^ Unlike many previous studies examining the association between breastfeeding duration and adiposity, the present study further adjusted for a number of other reproductive factors (e.g. age at menarche, parity and age at first birth), which may have contributed partly to the different association observed. Previously, there was little good evidence about the associations of adiposity with age at menopause or total reproductive years. In a few studies that reported relevant findings, nearly all did not consider other reproductive factors simultaneously, and the results have been inconsistent. A small US study of 522 post-menopausal women found no association between age at menopause and adiposity measurements.^16^ Similarly, in a recent report among 200 000 women in the UK Biobank study, there was no association between age at menopause and adiposity measurements.^7^ In a Chinese study of 1429 post-menopausal women, there was a positive association between adiposity and reproductive years, but the strength of the association was about half as strong as in the present study.^6^ With more detailed reproductive history information collected and comprehensive adjustment for potential confounders,^6^^,^^16^ our study of 128 000 post-menopausal women thus provides reliable new evidence of a strong positive association of adiposity with age at menopause and total reproductive years. Longer total reproductive years may reflect greater cumulative exposure to female hormones, leading to increased body fat accumulation.

The strength of this study includes its large sample size of both pre- and post-menopausal women, the good quality and completeness of data collected, and good reproducibility.^8^ A comprehensive range of information including both lifestyle factors and life-course reproductive factors allowed us to simultaneously control for potential confounders to reliably assess the independent association between reproductive factors and adiposity. Furthermore, there was high repeatability of the information collected (e.g. the intra-class correlation coefficients of 0.84 for age at menarche and 0.96 for number of live birth between the baseline survey and 5% randomly selected re-survey). In contrast to our study, most previous reports on adiposity and reproductive history tended to focus only on BMI, with limited data available on other measures of adiposity such as WC, %BF or WHR. Different measures of adiposity, though highly correlated with each other, may be associated somewhat differently with specific diseases.^20^^,^^21^ Several different measures of general and central adiposity were explored and, with few exceptions, each was shown to be associated with various reproductive factors. In our study, the greatly attenuated associations between WC and reproductive factors after additional adjustment for BMI suggested that, in this relatively lean Chinese population, general adiposity is more strongly associated with reproductive factors compared with central adiposity. An important limitation of our study is the lack of data on childhood adiposity, which may influence the observed association between age at menarche and adult adiposity. However, findings from a birth cohort of 3743 Scottish females suggested that pubertal timing was found to predict adult adiposity independently of childhood BMI, with adjustment for childhood BMI only accounting for about 10% of the association between age at menarche and adult BMI.^22^ Further evidence from genome-wide association studies in European women showed that multiple loci associated with age at menarche and menopause was also associated with adult BMI, indicating possible shared genetic pathways influencing these outcomes, but the causal directions between these traits were not clear.^23–25^ The present study used women’s reproductive history as the exposure and later adult adiposity as the outcome. However, adult adiposity may also affect certain reproductive characteristics in women (e.g. age at menopause). Indeed, mean age at menopause was slightly earlier at lower levels of adiposity (Supplementary Figure 4, available as Supplementary data at *IJE* online). This reverse association may complicate the relationship between age at menopause and adiposity. Genetic evidence in Chinese women is, however, limited and large-scale genome-wide studies may provide information on the biological and causal relationships between reproductive factors and adiposity in East Asians. Another limitation is the cross-sectional study design and use of self-reported reproductive factors. Although few women smoked or drank alcohol regularly and the adjustment for other lifestyle or reproductive risk factors was also considered in the analyses, residual confounding from other unknown risk factors may still remain.

This large study has provided robust new evidence that adiposity later in adult life was associated significantly with several life-course reproductive factors, particularly earlier age at menarche and at first birth, later age at menopause and longer total reproductive years. These results suggest that longer reproductive years providing prolonged exposure to hormones may result in greater fat accumulation. If these associations are largely or partly true, then, although the observed effects on adiposity of each factor may be modest, collectively, the overall effects may well be appreciable at the population level. Further detailed prospective and genetic investigations of the effects of reproductive factors, and the associated excess adiposity, on disease risks are warranted.

## Members of the China Kadoorie Biobank collaborative group

International Steering Committee: Junshi Chen, Zhengming Chen (PI), Rory Collins, Liming Li (PI), Richard Peto.

International Co-ordinating Centre, Oxford: Daniel Avery, Derrick Bennett, Yumei Chang, Yiping Chen, Zhengming Chen, Robert Clarke, Huaidong Du, Xuejuan Fan, Simon Gilbert, Alex Hacker, Michael Holmes, Andri Iona, Christiana Kartsonaki; Rene Kerosi, Ling Kong, Om Kurmi, Garry Lancaster, Sarah Lewington, John McDonnell, Winnie Mei, Iona Millwood, Qunhua Nie, Jayakrishnan Radhakrishnan, Sajjad Rafiq, Paul Ryder, Sam Sansome, Dan Schmidt, Paul Sherliker, Rajani Sohoni, Iain Turnbull, Robin Walters, Jenny Wang, Lin Wang, Ling Yang, Xiaoming Yang.

National Co-ordinating Centre, Beijing: Zheng Bian, Ge Chen, Yu Guo, Bingyang Han, Can Hou, Jun Lv, Pei Pei, Shuzhen Qu, Yunlong Tan, Canqing Yu, Huiyan Zhou.

10 Regional Co-ordinating Centres:

Qingdao—Qingdao CDC: Zengchang Pang, Ruqin Gao, Shaojie Wang, Yongmei Liu, Ranran Du, Yajing Zang, Liang Cheng, Xiaocao Tian, Hua Zhang. Licang CDC: Silu Lv, Junzheng Wang, Wei Hou.
Heilongjiang—Provincial CDC: Jiyuan Yin, Ge Jiang, Shumei Liu, Zhigang Pang, Xue Zhou. Nangang CDC: Liqiu Yang, Hui He, Bo Yu, Yanjie Li, Huaiyi Mu, Qinai Xu, Meiling Dou, Jiaojiao Ren.
Hainan—Provincial CDC: Jianwei Du, Shanqing Wang, Ximin Hu, Hongmei Wang, Jinyan Chen, Yan Fu, Zhenwang Fu, Xiaohuan Wang, Hua Dong. Meilan CDC: Min Weng, Xiangyang Zheng, Yijun Li, Huimei Li, Chenglong Li.
Jiangsu—Provincial CDC: Ming Wu, Jinyi Zhou, Ran Tao, Jie Yang. Suzhou CDC: Jie Shen, Yihe Hu, Yan Lu, Yan Gao, Liangcai Ma, Renxian Zhou, Aiyu Tang, Shuo Zhang, Jianrong Jin.
Guangxi—Provincial CDC: Zhenzhu Tang, Naying Chen, Ying Huang. Liuzhou CDC: Mingqiang Li, Jinhuai Meng, Rong Pan, Qilian Jiang, Jingxin Qing, Weiyuan Zhang, Yun Liu, Liuping Wei, Liyuan Zhou, Ningyu Chen, Jun Yang, Hairong Guan.
Sichuan—Provincial CDC: Xianping Wu, Ningmei Zhang, Xiaofang Chen, Xuefeng Tang. Pengzhou CDC: Guojin Luo, Jianguo Li, Xiaofang Chen, Jian Wang, Jiaqiu Liu, Qiang Sun.
Gansu—Provincial CDC: Pengfei Ge, Xiaolan Ren, Caixia Dong. Maiji CDC: Hui Zhang, Enke Mao, Xiaoping Wang, Tao Wang.
Henan—Provincial CDC: Guohua Liu, Baoyu Zhu, Gang Zhou, Shixian Feng, Liang Chang, Lei Fan. Huixian CDC: Yulian Gao, Tianyou He, Li Jiang, Huarong Sun, Pan He, Chen Hu, Qiannan Lv, Xukui Zhang.
Zhejiang—Provincial CDC: Min Yu, Ruying Hu, Le Fang, Hao Wang. Tongxiang CDC: Yijian Qian, Chunmei Wang, Kaixue Xie, Lingli Chen, Yaxing Pan, Dongxia Pan.
Hunan—Provincial CDC: Yuelong Huang, Biyun Chen, Donghui Jin, Huilin Liu, Zhongxi Fu, Qiaohua Xu. Liuyang CDC: Xin Xu, Youping Xiong, Weifang Jia, Xianzhi Li, Libo Zhang, Zhe Qiu.

## Supplementary Data

Supplementary data are available at *IJE* online.

## Funding

The CKB baseline survey and the first re-survey were supported by the Kadoorie Charitable Foundation in Hong Kong. The long-term follow-up is supported by the UK Wellcome Trust (088158/Z/09/Z, 104085/Z/14/Z), the Chinese Ministry of Science and Technology (2011BAI09B01) and the Chinese National Natural Science Foundation (81390541). The British Heart Foundation, the UK Medical Research Council and Cancer Research UK provide core funding to the Clinical Trial Service Unit and Epidemiological Studies Unit at Oxford University for the project.

## Supplementary Material

Supplementary Data
