# Supplementary material for: Adiposity in relation to age at menarche and other reproductive factors among 300 000 Chinese women: findings from China Kadoorie Biobank study
Source: Int J Epidemiol. 2016 Aug 12;46(2):502–12. doi: 10.1093/ije/dyw165 (PMC5837303; doi:10.1093/ije/dyw165)
Supplement: Supplementary Data [file dyw165_supp.docx]

**Webfigure 1: Proportions of women who had a) early age at menarche, b) nulliparous or c) late age at menopausal, by year of birth and area**

**Webfigure 4: Means of age at menopause by adiposity categories**

**
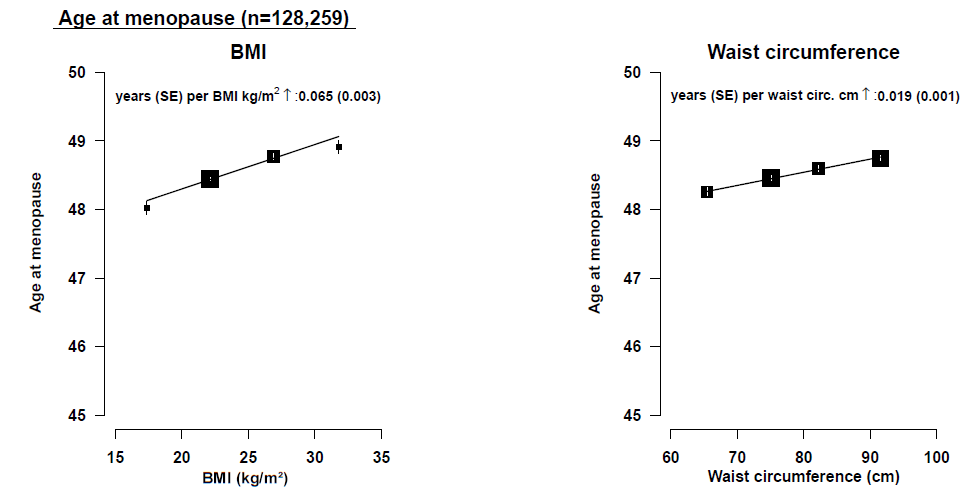
**

**Webtable 1: Socio-demographic and lifestyle characteristics by reproductive factors and adiposity**

|  | Birth cohorts | | Urban % | | High school or above,% | | Household income>20000 CNY/year | | Current regular smoker,% | | Weekly regular drinker,% | | Total physical activity, MET-h/day | |  |
| --- | --- | --- | --- | --- | --- | --- | --- | --- | --- | --- | --- | --- | --- | --- | --- |
|  | 1920s-30s | 1960s-70s |  |  |  |  |  |  |  |  |  |  |  |  |  |
| Age at menarche, years |  |  | |  | |  | |  | |  | |  | |  | |
| ≤12 | 4.8 | 57.9 | | 50.5 | | 27.1 | | 46.5 | | 1.7 | | 2.2 | | 22.4 | |
| 13-16 | 8.1 | 46.6 | | 43.5 | | 19.7 | | 42.1 | | 2.0 | | 1.9 | | 21.4 | |
| ≥17 | 12.7 | 20.4 | | 40.1 | | 10.8 | | 35.8 | | 2.9 | | 2.4 | | 19.5 | |
| Parity |  |  | |  | |  | |  | |  | |  | |  | |
| Nulliparous | 11.0 | 50.8 | | 63.9 | | 23.9 | | 35.4 | | 3.6 | | 2.6 | | 19.6 | |
| 1 or 2 children | 1.6 | 52.3 | | 48.4 | | 21.8 | | 44.2 | | 1.5 | | 1.9 | | 22.6 | |
| ≥3 children | 25.6 | 12.2 | | 30.3 | | 5.4 | | 29.6 | | 5.0 | | 2.5 | | 16.7 | |
| Age at first birth, years ^†^ |  |  | |  | |  | |  | |  | |  | |  | |
| ≤19 | 26.9 | 13.4 | | 19.1 | | 2.1 | | 27.6 | | 5.8 | | 2.9 | | 16.6 | |
| 20-25 | 7.8 | 44.6 | | 36.0 | | 15.3 | | 40.0 | | 2.3 | | 2.1 | | 21.4 | |
| ≥26 | 6.3 | 34.4 | | 73.1 | | 25.8 | | 43.8 | | 1.6 | | 1.9 | | 20.8 | |
| Breastfed per child, months ^†^ |  |  | |  | |  | |  | |  | |  | |  | |
| No feed | 5.1 | 53.3 | | 70.0 | | 27.0 | | 45.3 | | 1.6 | | 1.7 | | 20.8 | |
| ≤12 | 8.0 | 43.1 | | 56.2 | | 20.1 | | 42.7 | | 1.8 | | 1.9 | | 21.2 | |
| 13-24 | 10.5 | 36.8 | | 27.0 | | 13.6 | | 36.9 | | 2.6 | | 2.3 | | 20.3 | |
| ≥25 | 12.8 | 21.4 | | 16.1 | | 6.2 | | 29.1 | | 4.7 | | 2.9 | | 18.3 | |
| Age at menopause, years ^‡^ |  |  | |  | |  | |  | |  | |  | |  | |
| ≤42 | 27.5 | 8.3 | | 33.9 | | 8.7 | | 32.1 | | 4.8 | | 2.7 | | 17.0 | |
| 43-52 | 18.2 | 0.8 | | 44.3 | | 9.9 | | 38.0 | | 3.6 | | 2.4 | | 17.3 | |
| ≥53 | 19.2 | 0.0 | | 51.0 | | 9.5 | | 37.9 | | 3.4 | | 2.3 | | 16.4 | |
| Reproductive years, years ^‡^ |  |  | |  | |  | |  | |  | |  | |  | |
| ≤29 | 23.5 | 4.9 | | 36.4 | | 7.9 | | 33.3 | | 4.4 | | 2.6 | | 17.2 | |
| 30-35 | 18.1 | 0.6 | | 43.9 | | 9.4 | | 37.4 | | 3.6 | | 2.4 | | 17.3 | |
| ≥36 | 17.9 | 0.0 | | 51.5 | | 11.6 | | 40.4 | | 3.2 | | 2.3 | | 16.7 | |
| BMI, kg/m^2^ |  |  | |  | |  | |  | |  | |  | |  | |
| <18.5 | 19.5 | 33.2 | | 33.2 | | 17.8 | | 34.9 | | 5.4 | | 2.5 | | 19.9 | |
| 18.5-24.9 | 8.7 | 43.3 | | 40.8 | | 19.8 | | 41.3 | | 2.2 | | 2.1 | | 21.5 | |
| 25.0-29.9 | 8.8 | 34.0 | | 47.6 | | 15.2 | | 40.6 | | 1.9 | | 2.0 | | 20.3 | |
| ≥30 | 10.8 | 29.7 | | 51.6 | | 11.8 | | 39.4 | | 2.3 | | 1.7 | | 19.0 | |
| Waist circumference, cm |  |  | |  | |  | |  | |  | |  | |  | |
| <70 | 9.2 | 48.8 | | 37.5 | | 22.3 | | 38.2 | | 2.5 | | 2.0 | | 22.4 | |
| 70-79 | 7.2 | 46.1 | | 41.5 | | 20.6 | | 41.7 | | 2.0 | | 2.1 | | 21.9 | |
| 80-84 | 8.9 | 35.6 | | 44.8 | | 16.9 | | 41.9 | | 2.2 | | 2.1 | | 20.5 | |
| ≥85 | 12.8 | 25.6 | | 47.4 | | 12.0 | | 40.2 | | 2.5 | | 2.1 | | 19.0 | |

*^†^  Among parous women only.*

*^‡^ Among post-menopausal women only.*

**Webtable 2 Adjusted means (SD) of BMI, Fat percentages, WC and WHR, by reproductive factors**

| **Reproductive factors** | **BMI (kg/m^2^) *** | **%FAT *** | **WC (cm) *** | **WHR *** |
| --- | --- | --- | --- | --- |
| **Age at mena**r**che** |  |  |  |  |
| ≤12 | 24.5 (0.03) | 33.2 (0.06) | 80.2 (0.07) | 0.87 (0.00) |
| 13 | 24.2 (0.02) | 32.8 (0.04) | 79.6 (0.05) | 0.87 (0.00) |
| 14 | 24.0 (0.02) | 32.4 (0.03) | 79.2 (0.04) | 0.87 (0.00) |
| 15 | 23.8 (0.01) | 32.0 (0.03) | 78.7 (0.04) | 0.86 (0.00) |
| 16 | 23.6 (0.01) | 31.7 (0.03) | 78.5 (0.04) | 0.86 (0.00) |
| 17 | 23.4 (0.02) | 31.4 (0.03) | 78.2 (0.05) | 0.86 (0.00) |
| ≥18 | 23.1 (0.02) | 30.9 (0.03) | 77.5 (0.05) | 0.86 (0.00) |
| *Changes per menarche age ↑* | *-0.19 (0.003) -0.08(0.002)^$^* | *-0.33 (0.01)* | *-0.38 (0.009) 0.06(0.008)^$^* | *-0.002 (0.000)* |
| **Early menarche ( ≤ 12 years)** |  |  |  |  |
| No | 23.7 (0.01) | 31.8 (0.01) | 78.6 (0.02) | 0.86 (0.00) |
| Yes | 24.4 (0.03) | 33.1 (0.06) | 80.1 (0.07) | 0.87 (0.00) |
| **Ever had live birth ^#^** |  |  |  |  |
| Nulliparous | 23.4 (0.06) | 31.2 (0.12) | 77.7 (0.15) | 0.86 (0.00) |
| Parous | 23.7 (0.01) | 31.9 (0.01) | 78.7 (0.02) | 0.86 (0.00) |
| **Parity ^#^** |  |  |  |  |
| 1 | 23.5 (0.01) | 31.5 (0.03) | 77.8 (0.04) | 0.86 (0.00) |
| 2 | 23.9 (0.01) | 32.2 (0.02) | 79.1 (0.03) | 0.87 (0.00) |
| 3 | 23.8 (0.02) | 32.1 (0.03) | 79.2 (0.04) | 0.87 (0.00) |
| 4 | 23.8 (0.02) | 32.1 (0.05) | 79.5 (0.07) | 0.87 (0.00) |
| ≥5 | 23.7 (0.03) | 32.0 (0.06) | 79.4 (0.08) | 0.87 (0.00) |
| **Age at first birth (years) ^†^** |  |  |  |  |
| <20 | 24.0 (0.02) | 32.4 (0.05) | 79.4 (0.06) | 0.87 (0.00) |
| 20-21 | 23.9 (0.01) | 32.2 (0.03) | 79.1 (0.04) | 0.87 (0.00) |
| 22-23 | 23.8 (0.01) | 32.1 (0.03) | 78.9 (0.04) | 0.87 (0.00) |
| 24-25 | 23.6 (0.01) | 31.8 (0.03) | 78.5 (0.04) | 0.86 (0.00) |
| ≥26 | 23.5 (0.02) | 31.4 (0.03) | 78.0 (0.04) | 0.86 (0.00) |
| *Changes per first birth age↑* | *-0.05 (0.003) -0.01(0.001)^$^* | *-0.10 (0.01)* | *-0.12 (0.007) -0.008(0.004)^$^* | *0.000 (0.000)* |
| **Breastfed duration per child (months) ^†^** |  |  |  |  |
| ≤6 | 23.4 (0.03) | 31.5 (0.05) | 78.2 (0.07) | 0.86 (0.00) |
| 7-12 | 23.7 (0.01) | 31.9 (0.02) | 78.7 (0.03) | 0.87 (0.00) |
| 13-18 | 23.8 (0.01) | 32.1 (0.03) | 79.0 (0.04) | 0.87 (0.00) |
| 19-24 | 23.8 (0.02) | 31.9 (0.04) | 78.7 (0.05) | 0.87 (0.00) |
| ≥25 | 23.7 (0.03) | 31.9 (0.05) | 78.4 (0.07) | 0.86 (0.00) |
| **Age at menopause (years) ^‡^** |  |  |  |  |
| <43 | 23.5 (0.03) | 31.5 (0.07) | 79.5 (0.09) | 0.88 (0.00) |
| 43-47 | 23.6 (0.02) | 31.9 (0.04) | 79.8 (0.05) | 0.88 (0.00) |
| 48-50 | 23.8 (0.02) | 32.0 (0.03) | 80.0 (0.04) | 0.88 (0.00) |
| 51-52 | 24.0 (0.02) | 32.4 (0.05) | 80.7 (0.07) | 0.88 (0.00) |
| >=53 | 24.2 (0.03) | 32.8 (0.05) | 81.3 (0.07) | 0.89 (0.00) |
| *Changes per age at menopause↑* | *0.05 (0.002) 0.02(0.001)^$^* | *0.08 (0.01)* | *0.12 (0.007) -0.003(0.004)^$^* | *0.000 (0.000)* |
| **Late menopause ( ≥ 53 years) ^‡^** |  |  |  |  |
| No | 23.8 (0.01) | 32.0 (0.02) | 80.0 (0.03) | 0.88 (0.00) |
| Yes | 24.2 (0.03) | 32.8 (0.05) | 81.3 (0.07) | 0.89 (0.00) |
| **Reproductive years ^‡^** |  |  |  |  |
| <30 | 23.4 (0.02) | 31.4 (0.05) | 79.3 (0.06) | 0.88 (0.00) |
| 30-32 | 23.6 (0.02) | 31.8 (0.04) | 79.7 (0.05) | 0.88 (0.00) |
| 33-35 | 23.9 (0.02) | 32.3 (0.04) | 80.4 (0.05) | 0.88 (0.00) |
| >=36 | 24.3 (0.02) | 32.9 (0.04) | 81.3 (0.05) | 0.89 (0.00) |
| *Changes per reproductive year↑* | *0.07 (0.002) 0.02(0.001)^$^* | *0.12 (0.00)* | *0.17 (0.006) -0.01(0.003)^$^* | *0.001 (0.000)* |

***** Means were adjusted by age, study area, education, household income, smoking, alcohol drinking, physical activities and OC using. If the resulting categorical associations were linear, the change (SE) per unit exposure was estimated based on the multivariable linear regression model in which the continuous values of relevant reproductive factor were used (all P for trends <0.01); **^#^** Additionally adjusted for age at menarche in the model; **^†^** Among parous women only; additionally adjusted for age at menarche, parity, age at first birth and breastfeeding duration; **^‡^** Among post-menopausal women only; additionally adjusted for age at menarche (only for age at menopause), parity, age at first birth and breastfeeding duration; ^$^ After further adj. WC for BMI estimates and BMI for WC estimates

**Webtable 3 Adjusted means of BMI and WC by reproductive factors, by areas**

| **Reproductive factors** | **BMI (kg/m^2^) *** | | **WC (cm) *** | |
| --- | --- | --- | --- | --- |
|  | **Rural** | **Urban** | **Rural** | **Urban** |
| **Age at menarche** |  |  |  |  |
| ≤12 | 24.3 | 24.8 | 79.9 | 80.8 |
| 13 | 24.0 | 24.5 | 79.3 | 80.1 |
| 14 | 23.7 | 24.3 | 78.8 | 79.7 |
| 15 | 23.5 | 24.1 | 78.3 | 79.3 |
| 16 | 23.3 | 23.9 | 77.9 | 79.2 |
| 17 | 23.1 | 23.8 | 77.6 | 78.9 |
| ≥18 | 22.8 | 23.5 | 76.8 | 78.4 |
| *Changes per menarche age ↑* | *-0.21* | *-0.18* | *-0.45* | *-0.31* |
| **Early menarche ( ≤ 12 years)** |  |  |  |  |
| No | 23.4 | 24.0 | 78.1 | 79.3 |
| Yes | 24.2 | 24.7 | 79.8 | 80.6 |
| **Ever had live birth ^#^** |  |  |  |  |
| Nulliparous | 23.5 | 23.8 | 78.0 | 78.4 |
| Parous | 23.4 | 24.1 | 78.1 | 79.4 |
| **Parity ^#^** |  |  |  |  |
| 1 | 23.3 | 24.0 | 77.5 | 78.8 |
| 2 | 23.4 | 24.4 | 78.0 | 80.2 |
| 3 | 23.4 | 24.2 | 78.3 | 80.1 |
| 4 | 23.6 | 24.1 | 78.9 | 80.1 |
| ≥5 | 23.7 | 24.0 | 79.5 | 80.2 |
| **Age at first birth (years) ^†^** |  |  |  |  |
| <20 | 23.8 | 24.5 | 79.1 | 79.6 |
| 20-21 | 23.5 | 24.4 | 78.5 | 79.8 |
| 22-23 | 23.4 | 24.2 | 78.0 | 79.9 |
| 24-25 | 23.3 | 24.1 | 77.7 | 79.6 |
| ≥26 | 23.2 | 23.9 | 77.4 | 79.0 |
| *Changes per first birth age↑* | *-0.06* | *-0.05* | *-0.18* | *-0.09* |
| **Breastfed duration per child (months) ^†^** |  |  |  |  |
| ≤6 | 23.2 | 23.9 | 77.6 | 79.1 |
| 7-12 | 23.4 | 24.1 | 78.1 | 79.5 |
| 13-18 | 23.5 | 24.3 | 78.3 | 80 |
| 19-24 | 23.5 | 24.2 | 78.3 | 79.4 |
| ≥25 | 23.5 | 24.1 | 78.0 | 79.3 |
| **Age at menopause (years) ^‡^** |  |  |  |  |
| <43 | 22.9 | 24.2 | 78.1 | 81.3 |
| 43-47 | 23.1 | 24.3 | 78.6 | 81.5 |
| 48-50 | 23.3 | 24.4 | 78.9 | 81.4 |
| 51-52 | 23.5 | 24.6 | 79.6 | 82.1 |
| >=53 | 23.7 | 24.9 | 80.2 | 82.7 |
| *Changes per age at menopause↑* | *0.05* | *0.05* | *0.13* | *0.10* |
| **Late menopause ( ≥ 53 years) ^‡^** |  |  |  |  |
| No | 23.2 | 24.4 | 78.9 | 81.5 |
| Yes | 23.7 | 24.9 | 80.2 | 82.7 |
| **Reproductive years ^‡^** |  |  |  |  |
| <30 | 22.9 | 24.1 | 78.0 | 81.0 |
| 30-32 | 23.1 | 24.2 | 78.5 | 81.1 |
| 33-35 | 23.4 | 24.5 | 79.3 | 81.7 |
| >=36 | 23.8 | 24.9 | 80.3 | 82.6 |
| *Changes per reproductive year↑* | *0.08* | *0.07* | *0.18* | *0.14* |

***** Means were adjusted by age, study area, education, household income, smoking, alcohol drinking, physical activities and OC using. If the resulting categorical associations were linear, the change per unit exposure was estimated based on the multivariable linear regression model in which the continuous values of relevant reproductive factor were used (all P for trends <0.01).; **^#^** Additionally adjusted for age at menarche in the model ; **^†^** Among parous women only; additionally adjusted for age at menarche, parity, age at first birth and breastfeeding duration ; **^‡^** Among post-menopausal women only; additionally adjusted for age at menarche (only for age at menopause), parity, age at first birth and breastfeeding duration

**Webtable 4 Adjusted means of BMI and WC by reproductive factors, among pre- and post- menopausal women**

| **Reproductive factors** | **BMI (kg/m^2^) *** | |  | **WC (cm) *** | |
| --- | --- | --- | --- | --- | --- |
|  | **Pre/peri-menopause** | **Post-menopause** |  | **Pre/peri-menopause** | **Post-menopause** |
| **Age at menarche** |  |  |  |  |  |
| ≤12 | 24.4 | 24.5 |  | 78.7 | 81.7 |
| 13 | 24.0 | 24.4 |  | 78.0 | 81.4 |
| 14 | 23.8 | 24.2 |  | 77.5 | 81.0 |
| 15 | 23.5 | 24.0 |  | 77.0 | 80.6 |
| 16 | 23.4 | 23.8 |  | 76.8 | 80.3 |
| 17 | 23.2 | 23.6 |  | 76.6 | 79.8 |
| ≥18 | 23.0 | 23.3 |  | 76.1 | 79.1 |
| *Changes per menarche age ↑* | *-0.20* | *-0.18* |  | *-0.37* | *-0.40* |
| **Early menarche ( ≤ 12 years)** |  |  |  |  |  |
| No | 23.6 | 23.8 |  | 77.1 | 80.1 |
| Yes | 24.4 | 24.5 |  | 78.7 | 81.6 |
| **Ever had live birth ^#^** |  |  |  |  |  |
| Nulliparous | 23.4 | 23.6 |  | 76.4 | 79.5 |
| Parous | 23.6 | 23.8 |  | 77.3 | 80.2 |
| **Parity ^#^** |  |  |  |  |  |
| 1 | 23.5 | 23.4 |  | 76.8 | 78.7 |
| 2 | 23.7 | 23.9 |  | 77.7 | 80.2 |
| 3 | 23.8 | 23.9 |  | 78.1 | 80.4 |
| 4 | 23.9 | 23.9 |  | 78.5 | 80.8 |
| ≥5 | 23.9 | 23.9 |  | 78.7 | 80.9 |
| **Age at first birth (years) ^†^** |  |  |  |  |  |
| <20 | 24.2 | 24.2 |  | 78.7 | 81.2 |
| 20-21 | 23.8 | 24.0 |  | 77.8 | 80.6 |
| 22-23 | 23.7 | 23.8 |  | 77.4 | 80.3 |
| 24-25 | 23.6 | 23.7 |  | 77.1 | 80.0 |
| ≥26 | 23.4 | 23.4 |  | 76.8 | 79.2 |
| *Changes per first birth age↑* | *-0.05* | *-0.07* |  | *-0.13* | *-0.18* |
| **Breastfed duration per child (months) ^†^** |  |  |  |  |  |
| ≤6 | 23.3 | 23.7 |  | 76.6 | 79.9 |
| 7-12 | 23.6 | 23.8 |  | 77.2 | 80.3 |
| 13-18 | 23.8 | 23.9 |  | 77.6 | 80.5 |
| 19-24 | 23.8 | 23.8 |  | 77.7 | 80.1 |
| ≥25 | 23.8 | 23.8 |  | 77.6 | 79.8 |

***** Means were adjusted by age, study area, education, household income, smoking, alcohol drinking, physical activities and OC using. If the resulting categorical associations were linear, the change per unit exposure was estimated based on the multivariable linear regression model in which the continuous values of relevant reproductive factor were used (all P for trends <0.01); **^#^** Additionally adjusted for age at menarche in the model; **^†^** Among parous women only; additionally adjusted for age at menarche, parity, age at first birth and breastfeeding duration.

**Webtable 5 Adjusted means of BMI and WC by reproductive factors, among women with different level of education**

| **Reproductive factors** | **BMI (kg/m^2^) *** | | | | **WC (cm) *** | | | |
| --- | --- | --- | --- | --- | --- | --- | --- | --- |
|  | No formal school | Primary school | Secondary school | High school or above | No formal school | Primary school | Secondary school | High school or above |
| **Age at menarche** |  |  |  |  |  |  |  |  |
| ≤12 | 24.6 | 24.7 | 24.5 | 24.2 | 81.3 | 81.1 | 79.7 | 78.4 |
| 13 | 24.4 | 24.3 | 24.3 | 23.8 | 81.0 | 80.5 | 79.1 | 77.7 |
| 14 | 24.0 | 24.1 | 24.1 | 23.6 | 80.2 | 80.1 | 78.7 | 77.2 |
| 15 | 23.8 | 23.9 | 23.8 | 23.4 | 79.6 | 79.6 | 78.1 | 76.9 |
| 16 | 23.6 | 23.7 | 23.6 | 23.3 | 79.4 | 79.2 | 77.9 | 76.9 |
| 17 | 23.3 | 23.5 | 23.5 | 23.3 | 78.7 | 78.8 | 77.8 | 76.9 |
| ≥18 | 23.1 | 23.2 | 23.3 | 23.3 | 77.9 | 78.0 | 77.4 | 76.9 |
| *Changes per menarche age ↑* | *-0.22* | *-0.21* | *-0.19* | *-0.15* | *-0.50* | *-0.44* | *-0.34* | *-0.23* |
| **Early menarche ( ≤ 12 years)** |  |  |  |  |  |  |  |  |
| No | 23.6 | 23.7 | 23.8 | 23.5 | 79.2 | 79.3 | 78.2 | 77.1 |
| Yes | 24.5 | 24.6 | 24.5 | 24.2 | 81.3 | 81.0 | 79.6 | 78.4 |
| **Ever had live birth ^#^** |  |  |  |  |  |  |  |  |
| Nulliparous | 23.5 | 23.9 | 23.6 | 23.1 | 78.7 | 79.3 | 77.5 | 76.1 |
| Parous | 23.6 | 23.8 | 23.8 | 23.6 | 79.3 | 79.3 | 78.3 | 77.3 |
| **Parity ^#^** |  |  |  |  |  |  |  |  |
| 1 | 23.5 | 23.6 | 23.7 | 23.5 | 78.9 | 78.6 | 77.6 | 76.8 |
| 2 | 23.6 | 23.8 | 24.0 | 23.8 | 79.0 | 79.4 | 78.9 | 78.3 |
| 3 | 23.6 | 23.8 | 24.0 | 23.9 | 79.1 | 79.6 | 79.3 | 78.6 |
| 4 | 23.6 | 23.9 | 24.1 | 23.9 | 79.5 | 80.1 | 79.6 | 79.3 |
| ≥5 | 23.7 | 23.9 | 24.1 | 23.7 | 80.1 | 80.3 | 79.9 | 79.1 |
| **Age at first birth (years) ^†^** |  |  |  |  |  |  |  |  |
| <20 | 23.8 | 24.2 | 24.5 | 23.8 | 79.9 | 80.6 | 80.0 | 77.6 |
| 20-21 | 23.7 | 23.9 | 24.1 | 23.8 | 79.5 | 79.7 | 79.0 | 78.1 |
| 22-23 | 23.5 | 23.8 | 23.9 | 23.8 | 79.0 | 79.3 | 78.5 | 77.8 |
| 24-25 | 23.4 | 23.6 | 23.8 | 23.7 | 78.9 | 78.9 | 78.1 | 77.5 |
| ≥26 | 23.3 | 23.5 | 23.6 | 23.5 | 78.4 | 78.5 | 77.7 | 77.0 |
| *Changes per first birth age↑* | *-0.06* | *-0.08* | *-0.06* | *-0.03* | *-0.15* | *-0.21* | *-0.15* | *-0.08* |
| **Breastfed duration per child (months) ^†^** | |  |  |  |  |  |  |  |
| ≤6 | 23.5 | 23.6 | 23.6 | 23.2 | 79.0 | 79.0 | 77.8 | 76.5 |
| 7-12 | 23.6 | 23.7 | 23.8 | 23.5 | 79.3 | 79.3 | 78.2 | 77.2 |
| 13-18 | 23.7 | 23.9 | 24.0 | 23.8 | 79.6 | 79.6 | 78.5 | 77.7 |
| 19-24 | 23.5 | 23.8 | 24.0 | 24.0 | 79.1 | 79.4 | 78.8 | 78.2 |
| ≥25 | 23.5 | 23.8 | 24.0 | 24.2 | 78.8 | 79.2 | 78.5 | 78.5 |
| **Age at menopause (years) ^‡^** |  |  |  |  |  |  |  |  |
| <43 | 23.1 | 23.5 | 24.0 | 23.9 | 78.5 | 80.0 | 80.7 | 80.2 |
| 43-47 | 23.3 | 23.7 | 24.1 | 24.0 | 79.1 | 80.2 | 80.7 | 80.1 |
| 48-50 | 23.4 | 23.8 | 24.3 | 24.0 | 79.2 | 80.6 | 80.8 | 79.8 |
| 51-52 | 23.8 | 24.0 | 24.6 | 24.3 | 80.2 | 81.0 | 81.4 | 80.5 |
| >=53 | 23.9 | 24.3 | 24.7 | 24.5 | 80.5 | 81.8 | 81.8 | 81.1 |
| *Changes per age at menopause↑* | *0.05* | *0.05* | *0.05* | *0.04* | *0.12* | *0.13* | *0.08* | *0.06* |
| **Reproductive years ^‡^** |  |  |  |  |  |  |  |  |
| <30 | 23.1 | 23.4 | 23.9 | 23.8 | 78.5 | 79.7 | 80.4 | 80.0 |
| 30-32 | 23.3 | 23.6 | 24.0 | 23.9 | 78.9 | 80.2 | 80.5 | 79.6 |
| 33-35 | 23.6 | 24.0 | 24.4 | 24.0 | 79.7 | 80.9 | 81.1 | 79.9 |
| >=36 | 24.0 | 24.4 | 24.7 | 24.5 | 80.8 | 81.8 | 81.7 | 80.8 |
| *Changes per reproductive year↑* | *0.07* | *0.08* | *0.07* | *0.06* | *0.18* | *0.18* | *0.13* | *0.09* |

***** Means were adjusted by age, study area, education, household income, smoking, alcohol drinking, physical activities and OC using. If the resulting categorical associations were linear, the change per unit exposure was estimated based on the multivariable linear regression model in which the continuous values of relevant reproductive factor were used(all P for trends <0.01); **^#^** Additionally adjusted for age at menarche in the model; **^†^** Among parous women only; additionally adjusted for age at menarche, parity, age at first birth and breastfeeding duration; **^‡^** Among post-menopausal women only; additionally adjusted for age at menarche (only for age at menopause), parity, age at first birth and breastfeeding duration
